# Policy and discursive shifts in China’s economic diplomacy amid rising geopolitical tensions

**DOI:** 10.12688/openreseurope.23140.1

**Published:** 2026-04-13

**Authors:** Gábor Szüdi, Frank Gaenssmantel, Carmen Amado Mendes, Philipp Brugner

**Affiliations:** 1 Centre for Social Innovation (ZSI), Vienna, Vienna, Austria; 2 University of Groningen, Groningen, Groningen, The Netherlands; 3 President of the Macau Scientific and Cultural Centre (CCCM), Lisbon, Lisbon, Portugal

**Keywords:** China, European Union, Economic diplomacy, De-risking, Discursive representation, Leadership speeches

## Abstract

**Background:**

China seems to have entered into a new global leadership phase of economic diplomacy where its actors increasingly resort to new resources, tools and strategies serving well-defined national interests, such as the Made in China 2025 initiative, the Dual Circulation Strategy (DCS) and the Belt and Road Initiative (BRI). This has intensified the interrelated challenges to the EU’s economic security, industrial competitiveness and strategic autonomy, contributing to increasing supply chain vulnerabilities and dependencies. This increased economic challenge resulted in more assertive multi-layered EU-level policy approach, characterized by the de-risking strategy with four complementary policy goals.

**Methods:**

We used deductive coding of meticulously selected leadership speeches by Chinese top policymakers between 2013 and 2022 to assess whether this shift in EU-China economic diplomacy relations had an impact on the key narratives within the official Chinese high-level policy statements. The coding focuses on the significant and meaningful representation of the EU by China and vice versa, and on particular positive or negative descriptions of the EU by China and vice versa.

**Results:**

Our main findings entail that, despite the more grounded EU approach towards China, no significant alterations are apparent in Chinese leadership discourse which remains at a pragmatic level focused on mutual benefits as long as economic engagement affairs are not interlinked with Chinese governance or territorial issues.

**Conclusions:**

These findings suggest that China needs the EU as a reliable economic partner amid the growing geostrategic tensions towards the US or Japan, which provides the EU with a strategic room for manoeuvre towards open strategic autonomy. Such autonomy could be strengthened by re-negotiating core aspects of economic links with China, such as the relocation of more productive facilities of Chinese green technologies to Europe or pushing for a more open attitude from China towards European investments in critical economic sectors.

## Plain Language summary

This article looks at how China’s approach to economic diplomacy has changed in recent decades, especially towards Europe.

We see that China now relies more on big national strategies like the Belt and Road Initiative (BRI) or Made in China 2025 initiative. The main aim of these strategies is to make China’s economy stronger and more independent, therefore they create political and economic problems for the EU, such as greater dependence on Chinese supply chains and risks to Europe’s economic security and industrial strength.

In response, the EU has also changed its official policy, dubbing China a “systemic rival” and adopting a strategy to reduce risks. This includes strengthening EU industries, using trade protection tools, protecting critical sectors and working more closely with like-minded partners.

In spite of the changed European rhetoric, China’s official language has not changed much and still stresses cooperation and mutual benefits. This is true until the EU does not delve into issues perceived sensitive by China, such as governance or territorial questions.

Overall, the article argues that the EU is still not powerless and has room to rethink and reshape its economic relationship with China.

## Introduction

This research paper tries to assess – through coded analysis of Chinese leadership speeches – whether the interrelated and mutually reinforcing recent changes in China’s and the European Union’s (EU) economic diplomacy had an impact on the tone and narratives of official Chinese high-level policy expressions. Before the presentation of the methods used and results obtained from the coding, the Introduction aims to provide historical context by describing the different phases of the Chinese economic diplomacy, arriving at its modern stage which is characterized by three main national policy instruments operationalising China’s key economic goals. Then we shortly present the de-risking strategy and its related key measures introduced by the EU to counter the political-economic challenges – manifested in supply chain vulnerabilities and dependencies – exacerbated by the Chinese policy instruments.

### The modern era of Chinese economic diplomacy: the push for global leadership

For the purposes of this article, we understand economic diplomacy as “decision-making and negotiation in international economic relations” ( Bayne & Woolcock, 2016) for which all available economic tools and resources can be utilised to achieve national interests ( Chohan, 2021), including among others policy decisions to influence trade relations, exports, imports, investments, lending or aid ( van Bergeijk & Moons., 2009).

Aligned with this broad definition, we argue that a noticeable shift can be observed in the official Chinese economic diplomacy in recent decades, characterised by more authoritative policy norms and instruments that challenge the EU and other global like-minded partners in fundamental ways. The main economic goal of China is not global (re-)integration any longer but the build-up of an unchallenged leadership position which also offers a counter-narrative to the traditional Western economic and trade models.

The Chinese policymakers have realised the strategic importance of economic diplomacy in foreign strategy early after the declaration of the People’s Republic of China (PRC) in 1949 but – due to the undeveloped economic realities and the global political isolation of the new Communist state – the relevant actions remained extremely limited both in scope and intensity. ( Li, 2022) As the PRC’s official economic policy went through a paradigm shift with the gradual opening up of the country, the methods and goals of economic diplomacy also fundamentally changed.

China’s economic diplomacy always served its stated national interests that reflect the evolving relationship between the PRC and the outside world. In this framework, China’s economic diplomacy can be divided into five stages ( He, 2019): (1) *isolation* (1949–1978) when China was mostly disengaged from other countries; (2) *engagement* (1978–1989) when the country re-started its foreign trade after abandoning the Maoist policy of economic self-reliance for the development of productive forces under Deng Xiaoping; (3) *integration* (1989–2001) when China was successfully re-integrated into the global economic and trade system, culminating in the country’s entry to WTO in 2001; (4) *participation* (2001–2008 or 2012) ^1^ when the PRC put economic issues at the service of diplomacy, characterised by a newly intensive participation in the international economic system, along with the established global powers; (5) *leadership* (since 2008/12 –) when – in the wake of its newfound economic power and in the name of security and control – the PRC put forth a series of strategic notions on economic diplomacy that would put China at the centre of the international order.

This new Chinese economic vision – that can be interpreted from a Western perspective as an alternative to the current multilateral system – have increasingly led to tensions with other key actors, notably the United States and the European Union ( Li and Sun, 2014). In its modern incarnation the Chinese economic diplomacy has become more assertive and aimed to turn hard power to soft power by offering alternative narratives to Western economic and trade policies in addition to economic carrots and sticks. China’s soft power initiatives have become more global, with growing influence in Africa, Asia and Latin America through investments in economic development and infrastructure ( Rafiq, 2025). Since 2013, the infrastructure projects are mainly implemented under the *Belt and Road Initiative* (BRI) providing the means and locations (global infrastructure) to sell high-tech products and services according to the Chinese central strategies of domestic technology ambitions, with the aim of fostering economic interdependence and expanding China’s geopolitical influence by integrating other countries into its economic, trade and technological ecosystem.

### The national strategies behind China’s contemporary economic diplomacy

The nationalistic objectives of China’s new period of economic diplomacy are operationalised through a suite of integrated national strategies – notably the *Made in China 2025 initiative*, the *Dual Circulation Strategy (DCS)* and the *Belt and Road Initiative (BRI)* – which collectively exert pressure on the EU’s core economic sectors, create critical supply chain vulnerabilities and extend China’s geopolitical influence into the European neighbourhood. As such, this section analyses the multifaceted challenge posed by China to the EU’s economic security, industrial competitiveness and strategic autonomy.

The primary vectors of economic challenge stem from China’s assertive, state-led industrial policy aimed at achieving global dominance in high-technology sectors. The Made in China 2025 initiative serves as the roadmap for this transformation, seeking to elevate China from a labour-intensive manufacturer to a global leader in key high-tech areas, including robotics, aviation and new energy vehicles ( Kuo, 2025; Wübbek et al., 2016). This initiative is characterised by massive state subsidies, opaque public funding, and the strategic goal of domestic sourcing and indigenous innovation, often at the expense of fair market competition ( Liu et al., 2022). The resulting resource misallocation and targeted state support have further contributed to significant industrial overcapacity in China, particularly in “green” technologies that are critical for the EU’s own decarbonization goals.

The Electric Vehicle (EV) sector is the most prominent current example, encapsulating the systemic friction between the two economic models. China has consolidated its position as the global powerhouse in EV and battery production, manufacturing approximately 60% of the world’s EVs and 80% of its batteries ( IEA, 2025; Mazzocco, & Featherston, 2025). This dominance, fuelled by years of successful industrial policy and state subsidies, has given Chinese EV manufacturers a substantial cost advantage, estimated to be up to 25% lower than their European competitors ( Leggett, 2025; Osthoff & Goodman, 2025). This cost disparity, combined with declining domestic demand in China, has resulted in a surge of subsidised Chinese-made EVs entering the highly open European market, threatening to cause “material injury” to the foundational European automotive industry ( Featherston, 2024).

Complementing *Made in China 2025* is the *Dual Circulation Strategy (DCS*), adopted by Beijing to enhance self-reliance and technological resilience against external shocks ( García-Herrero, 2021). The DCS prioritises “internal circulation” – bolstering domestic production and consumption – while maintaining “external circulation” through selective, strategic engagement with the global economy. For the EU, the DCS presents a double risk: domestically, it pushes for import substitution, effectively making the Chinese market less accessible to high-value European goods (e.g., intermediate components and high-end machinery); externally, it accelerates the export of China’s massive industrial overcapacity into third markets, including the EU, as a means of sustaining domestic growth and mitigating internal economic slowdowns ( EC, 2025a). This strategy thus actively undermines the principle of reciprocal market access, forcing European companies to compete against state-backed Chinese rivals not only globally, but increasingly within the European single market itself.

Overall, the geopolitical dimension of China’s rise is encapsulated in the *BRI*, a massive infrastructure development and investment strategy that has evolved into a tool for projecting strategic influence and creating dependencies. While initially presented as a benign connectivity project, the BRI’s activities globally, including in Europe and its neighbourhood, often lack transparency and adherence to international standards, leading to concerns about debt sustainability and strategic control ( Calabrese, 2025).

A concrete example of the BRI’s strategic impact on Europe is China’s acquisition or partial control of key logistical infrastructure within the continent. The investment in the Piraeus Port in Greece, transforming it into a major regional logistics hub and a key point of entry for Chinese goods into Europe, provides Beijing with strategic footholds and leverage over European supply chains ( Kotoulas, 2024). Similar partial acquisitions or investments have occurred in other critical ports across Belgium, the Netherlands, Spain, Croatia and Italy ( Budak, 2025; Jacobs, 2023). These investments in strategic assets within EU member states raise concerns regarding potential control over communications systems, cyber espionage risks and the ability to influence EU foreign policy positions by capitalising on existing economic leverage over recipient states.

Furthermore, the BRI has been criticised for creating unsustainable debt burdens in vulnerable nations, often termed “debt-trap diplomacy” ( Chellaney, 2017; Brautigam, 2019; Jones & Hameiri, 2020) ^2^. The controversial Chinese-built highway project in Montenegro, for instance, significantly increased the country’s debt load, illustrating how stringent loan-repayment conditions and opaque financing models can expose already fragile economies to fiscal instability and over-dependence on Beijing ( Euractiv, 2021; Muller, 2024). The proliferation of the *Digital Silk Road* – the component of the BRI focused on digital infrastructure and emerging technologies – poses an additional systemic challenge by exporting elements of China’s techno-authoritarian governance model, such as facial recognition software and surveillance systems, to partners like Serbia ( Janković & Standish., 2025; Krivokapić, 2022). This not only extends China’s technological reach but also challenges the democratic principles of privacy and governance in the EU’s immediate vicinity.

### The impact of China’s contemporary economic diplomacy on the EU: supply chain vulnerabilities and dependencies

Beyond the direct challenge to industrial competitiveness, China’s centralised economic model creates profound supply chain vulnerabilities and dependencies for the EU across strategically vital sectors. The reliance on China for Critical Raw Materials (CRMs) and Active Pharmaceutical Ingredients (APIs) represents a significant non-market risk that challenges the EU’s ambition for strategic autonomy and exposes it to potential economic coercion ( GlobalData, 2024; Ragonnaud, 2024).

For instance, the dependence on CRMs, which are essential for the EU’s green and digital transitions (e.g., electric vehicle batteries, wind turbines, semiconductors), is acute. While CRMs are globally dispersed, China holds a dominant position in the crucial mid-stream stage: refining and processing ( Glaser and Naas, 2025). Specifically, the EU is dependent on China for an overwhelming majority of its rare earth element (REE) imports, and the reliance on specific materials like Magnesium stands at approximately 96% of all EU imports ( Banin et al., 2025; Corlin, 2025). China’s actions to leverage this dominance, such as imposing export restrictions on strategic materials like gallium and graphite in 2023, illustrate the geopolitical nature of these dependencies ( Huld, 2025; Reuters, 2025; Zimmermann, 2025). The concentration of production and processing within a single, strategically motivated jurisdiction enables China to exert significant leverage, effectively weaponizing supply chains for economic or political ends.

A similar, though often less publicised, vulnerability exists in the pharmaceutical sector. While Europe produces many APIs for innovative medicines domestically, a significant proportion of APIs for generic medicines and the raw chemical materials required for both innovative and generic drug production are overwhelmingly sourced from China and India ( EC, 2025a; GlobalData, 2024). The COVID-19 pandemic sharpened the EU’s awareness that a disruption in this supply chain, whether stemming from force majeure as in the case of the pandemic or from geopolitical conflict or economic coercion, could pose a major health and security crisis ( Gaub, 2020).

### The European response to China’s contemporary economic diplomacy: de-risking and its four pillars

The more assertive Chinese economic diplomacy made the EU rethink its official stance towards the Chinese government. This policy shift was formally acknowledged in 2019 when the EU defined a more critical, multi-faceted approach towards its relations with the PRC, characterising China not only as a cooperation and negotiating partner but also as an economic competitor and systemic rival ( EC, 2019b; EEAS, 2023). After decades of consensus on broad and expanding, mutually beneficial economic links with China, this language signaled a significant shift in European economic diplomacy, reflecting increasing concerns over trade imbalances, dependencies and vulnerabilities, lack of reciprocity and the violation of fundamental principles of competition and transparency.

In March 2023, *de-risking* became the official objective of the EU-China (economic) relations. In a keynote speech, Commission President von der Leyen claimed that the new Chinese strategies to steer the economy mean *“that the imperative for security and control now trumps the logic of free markets and open trade”,* which necessitates to “ *rebalance this relationship on the basis of transparency, predictability and reciprocity”* ( EC, 2023a). She called for an economic de-risking strategy – based on risk assessment and stress-testing – across four pillars: (1) making the EU economy and industry more competitive and resilient, (2) better using the existing EU toolbox of trade instruments, (3) developing new defensive tools for critical sectors and (4) seeking closer alignment with partners around the world ( MERICS, 2023).

In order to fulfil the objective of the first pillar, the EU introduced a set of policies aiming to make its economy more competitive and resilient. The growing dependencies on critical raw materials necessitated a coordinated European strategy, exemplified by the *Critical Raw Materials Act (CRMA)* in 2024, which was coupled with efforts to reinvest in domestic extraction, refining and recycling capacities, along with diversifying partnerships with non-Chinese suppliers ( EC, 2025b). The *Net Zero Industry Act* (NZIA) also aimed to reduce the unilateral dependence on Chinese suppliers for critical clean energy components and vowed to make the EU able to domestically produce at least 40% of its strategic net-zero technology needed for the Green Deal, such as solar, onshore and offshore wind, batteries and storage, heat pumps and grid technologies ( EC, 2025a). The *European Chips Act* also aims to promote European production in a critical technology area, namely semiconductors, thus promotes higher independence from Chinese suppliers and the timely availability of critical components within the EU ( EC, 2023b). By providing an exemption to semiconductors from the state aid ban, the Chips Act enables innovative subsidies to support the EU-wide development and expansion of advanced semiconductor plants, as well as investment into research and development ( Bandemer et al., 2025).

In addition to these novel acts, it is not clear what will happen to the stalled negotiations around the *Comprehensive Agreement on Investment* (CAI). China’s decision to impose sanctions on members of the European Parliament, along with a series of legal entities, essentially made it impossible for the European Parliament to continue its work towards the ratification of CAI. CAI is of utmost importance since it could address, at least in part, the decade-long key problem of overcoming “ *the persistence of market access obstacles in China*” as already stated in a 2003 EC document ( EC, 2003).

With regard to the existing EU toolbox of trade instruments that can be relevant also in connection to economic security concerns, several had been in place already before the start of the de-risking strategy in 2023. These measures should be efficiently implemented to counter economic distortions and threats posted by China. In response to the rapidly growing sales of subsidised Chinese-made EVs the EU initiated an anti-subsidy probe which led to the imposition of countervailing duties ranging up to 38% on Chinese EV imports, which underscores the seriousness of this competitive threat ( Labiak, 2024; Grieger, 2023). A new trade instrument is the *anti-coercion instrument (ACI)* ( EC, 2023c) which enables the EU to use import tariffs and other measures (import or export licenses, restrictions in service procurements). The countering of such external threats is coupled with addressing subsidized investments in the EU under the *Foreign Subsidies Regulation (FSR*) ( EC, 2022) which helps assess intra-EU cases such as Hungary’s subsidies granted to BYD but also addresses cases of subsidies by third-country (non-EU) public entities ( Tagliapietra et al., 2025).

The 2019 *Investment Screening Framework (ISF)* ( EC, 2019c) established a Cooperation Mechanism for Foreign Direct Investments (FDI) to support EU Member States (MS) in addressing economic security risks stemming from FDI. MSs and the Commission jointly review FDIs potentially affecting security, engaging in information exchange and raising concerns, if needed, on e.g. critical technology, infrastructure or data. Increasing China’s FDI certainly provided a stimulus to adopt the ISF, and its reference to “ *state-led outward projects or programmes*” is generally read as a reference to the BRI. At the same time, its actual screening activities are not predominantly aimed towards Chinese investors, also targeting trade partners from Western countries such as the US and UK ( Peragovics & Szunomár, 2025).

The *EU Toolbox for 5G Security* ( EC, 2020) was adopted in 2020. While the toolbox encourages cooperation among EU MSs to mitigate potential risks related to 5G, it does not entail legally binding obligations ( Bandemar et al., 2025). This resulted in a varying level of implementation among EU MSs: even though the toolbox recommended restrictions on “high-risk” suppliers such as Huawei or ZTE, some countries failed to introduce effective measures. There is however a trend towards stricter 5G security measures following the recent Chinese interference into telecommunications networks and the alleged bribery of Members of the European Parliament linked to Huawei ( Kroet, 2025).

As regards new defensive tools for critical sectors, the EU started to re-shape its future relationship with China in sensitive high-tech areas such as microelectronics, quantum computing, robotics, artificial intelligence (AI) or biotech through the new *Economic Security Strategy.* ( EC, 2023d). The Strategy and its follow-up recommendation ( EC, 2023e) mapped out where economic security needs to be strengthened and how trade defence tools could be improved. 10 critical technology areas were identified for joint risk assessment by the EC and EU MSs, out of which four areas are to be done urgently, namely in advanced semiconductors, AI technologies, quantum technologies and biotechnologies. The urgency of the risk assessment is justified by their potentially high exposure to foreign interference and the related risks of technology security infringement and technology leakage. The risk assessment led to an EU recommendation on screening outbound investment ( EC, 2025c) in three critical technologies (semiconductors, AI, quantum technologies) where investment can result in the development of military capabilities that pose risks to EU and national security. This entails that where dual-use purposes cannot be excluded there needs to be clear guidance or rules on whether investments or exports are in the security interests of the EU or EU MSs.

Finally, concerning greater alignment with other partners, the results are mixed: the EU-US Trade and Technology Council ceased its activities after the second inauguration of Donald Trump, while the second meeting of the EU-India Trade and Technology Council successfully took place in 2025 ( Indian Ministry of External Affairs, 2025), and the joint statement following the EU-Japan Summit in 2025 reiterated the need to promote enhanced cooperation based on the EU-Japan Green Alliance, the EU-Japan Partnership on Sustainable Connectivity and Quality Infrastructure and the EU-Japan Digital Partnership ( EC, 2025d). The strengthened economic partnership with India and Japan will enhance cooperation on sectors such as digital and clean technologies, also mitigating dependencies and vulnerabilities towards China. In addition, the EU was successfully entering into a new free trade agreement with New Zealand ( EC, 2024b), while the negotiations with India, Mexico and the Mercosur are in advanced stages.

The EU has also continued its investment into infrastructure in developing countries through the *Global Gateway strategy*, ( EC, 2021) offering an alternative to the BRI in terms of investment and finance. Countering China’s BRI, the EU aims to move from development-centred aid towards a more geostrategic use of investment tools where long-term, mutually beneficial partnerships are built with developing countries that align their priorities with European interests. In financial terms, the Global Gateway is a success, surpassing its initial development goal of EUR 306 billion in investment by October 2025 (with a new goal to mobilise a total amount of EUR 400 billion by 2027) ( Bilal, 2025).

## Methods

We aim to explore the potential discursive changes among Chinese policymakers towards the EU in the last decade that might be related to the shifting economic diplomacy relations as described in the Introduction section. Our main method is to use deductive coding on Chinese leadership speeches to gain a sophisticated picture on the Chinese high-level policy representation of the EU and its policies relevant to economic diplomacy.

We examined representations of the EU as an economic partner in leadership speeches and similar statements by Chinese top leaders between 2013 and 2022. The discussion relies on findings from the research project Changing Representations of the Other in China-EU Economic Relations—Tracing Discourses on the “Economic Other” through Leadership Speeches (or the Economic Other Project, in short), implemented at the University of Groningen with support from the ReConnect China consortium ^3^. The Economic Other Project includes speeches from the President, the prime minister, the minister of foreign affairs and the minister of commerce across the period from 2013 to 2022, i.e. the full first two terms of office of Chinese President Xi Jinping. Speeches were selected from official Chinese-language websites of the Chinese government bodies, most importantly the State Council website ( www.scio.gov.cn), with substantive statements on the EU as an international partner as main selection criterion. This means that speeches on non-EU related topics, where reference to the EU is limited and typically embedded within wider categories of international actors (like developed economies, destination of migration flows, etc.) were generally excluded. For the full list of selected speeches please refer to our dataset at Zenodo ( Gaenssmantel, 2026).

This coverage allows us to trace Chinese approaches towards the EU across a period of heightening bilateral tensions, stretching on the EU side from the Juncker Commission to the first three years under von der Leyen until just before the announcement of the de-risking strategy. It includes the moment when the EU shifted to the multi-faceted labelling of China as a partner, economic competitor and systemic rival in 2019 highlighted in previous sections. Since the project also addresses EU leadership discourse, we are able to comment on Chinese reactions to European criticisms as well.

Methodologically, the coding of leadership speeches started from a set of “expected representations”, extracted from academic commentary, policy analyses and news media on both the Chinese and the European side, including for example characterisations of the respective other as protectionist in either the commercial or investment spheres, as unfair trader or investor, or as complementary partner in technical spheres. To better grasp the role of economic engagement with the counterpart against major issues on the bilateral agenda, we also traced discourse on cooperation on climate change cooperation and within the BRI. Aside from this deductive dimension, the project also worked inductively to pick up major themes or characterisations of the counterpart that appeared prominently in the discourse beyond our specific expectations, such as the counterpart’s respect for economic governance rules. On top of concrete representations of the counterpart we also implemented more general codes on various degrees of negative or positive views on the counterpart, both in the economic sphere, but also beyond it. For the detailed coding system, please see our dataset on Zenodo ( Gaenssmantel, 2026).

As a caveat we must mention that leadership discourse is a challenging source. The collective mindset of policy makers is essentially inaccessible, in China just as anywhere else in the world, but the careful study of publicly available material can provide some clues on underlying ideas and intentions ( Cao, 2024); Nie, 2024). On the one hand, in China such speeches are carefully crafted, with no room for spontaneous, emotional expressions that could reveal “genuine” opinions or preferences of the speaker. But it also takes the listeners to the very core of the creation of China’s foreign policy discourse, namely to the “strategy makers [who] discursively construct a macro-narrative scenario of the context of the times and the international situation”. ( Song, 2022). In addition, the very fact that it is highly moderated confers a certain authoritativeness. After all, leadership discourse reflects input from scores of high officials reaching out to various groups within the foreign policy system, thus representing a pragmatic consensus across key players at the top of the political process. Also, policymakers are by no means isolated. They are at the heart of what has been termed a “discourse coalition,” comprising also official media and academics. ( Zhang & Orbie, 2021). The influence of think tank researchers on central foreign policy making is also well-established ( Xin, 2023).

## Results

China’s economic diplomacy entered a new phase in recent decades with a policy push towards global leadership, represented by comprehensive national strategies for pursuing China’s economic interests, as well as a bolder, more strategic rhetoric offering a new economic vision for the country’s partners and competitors. As seen in the Introduction section, this resulted in the recalibration of the EU’s economic strategy with the introduction of de-risking as the overarching goal. Through the above-described methodology we aimed to find out how the Chinese leaders represent the EU as an economic partner in this more disruptive period characterised by evolving tensions and conflicts, and whether it is possible to observe any significant changes in Chinese discourse over the past decade.

In the following paragraphs, we will highlight a few major trends that emerge from this extensive engagement with Chinese leadership discourse on the EU as an economic counterpart. When looking at the coding of Chinese leadership speeches across the years, what first hits the eye is the continuous dominance of positive, cooperative themes and the almost complete absence of representations of the EU as a problematic partner. With some fluctuations, we see a strong inclination of Chinese leaders to represent the EU as an important partner for technical cooperation in general, and more specifically for infrastructure and connectivity initiatives, both within and outside the Belt and Road Initiative (BRI) (even though towards the end of the period covered there is a decline of BRI-themed representations, clearly in reaction to more critical views on the initiative from actors within the EU).

An example are Prime Minister Li Keqiang’s comments at the Sixth China-CEEC Economic and Trade Forum in November 2016: “ *China has superior equipment and production capacity, mature, with good technology level and service systems and high cost performance; Central European countries have a need to speed up their industrialisation; developed countries in Western Europe have the most advanced technology and management experience; combing the respective advantages of the three sides will lead to considerable gains for all sides.*” ( Li, 2016). Later we see various examples of Foreign Minister Wang Yi emphasising the benefits of China-EU green and digital partnerships ( Wang, 2021a; Wang, 2022a). We also see frequent general references to the many areas of shared interests and agreement on international matters, as well as variations on the idea of “ *seeking commonalities, while reserving differences.”* ( Li, 2017; Wang 2021b; Wang 2021c).

References to the considerable potential of China-EU technical partnership peaked twice, in 2016 and 2020–2021. These peaks coincide with periods of sustained EU criticism of China, in the first phase in relation to overcapacity in steel production, in the second phase on barriers to EU investment in China and the absence of a level playing field during the final months of the negotiations on the CAI. This might suggest a tendency to counter criticism by emphasising the positive potential of economic cooperation.

The concrete representations most commonly associated with critical reporting on the EU in Chinese official news media, e.g. protectionism and discriminatory practices, are rarely visible in leadership speeches. But when they come, they are connected to specific EU policies or policy debates that are considered problematic. Thus in 2016, Li Keqiang indirectly criticises the EU for its hesitance about recognising China as a market economy and asks for respect for international commercial law in this regard. He also highlights concerns about fairness for Chinese investors amidst the debate on an EU investment screening mechanism ( Li, 2017). In 2019, Foreign Minister Wang Yi indirectly denounces European bans on Huawei’s participation in the construction of 5G infrastructure by calling for fair and non-discriminatory treatment of all firms, including those from China ( Wang, 2019a; Wang 2019b).

While the specific representations of the EU in the coding of Chinese leadership speeches fluctuate across time, no particular trend emerges. This suggests that EU policies or attitudes that are deemed unfavourable to China do not seem to have pushed representations of the EU by Chinese leaders in any particular direction. The same is true for most of the broader coding on positive and negative references to the EU. Across the entire period under study here, Chinese leaders bestowed generous praise upon the EU, both indirectly and directly, and both in the economic sphere and beyond. This includes for example comments on the “ *solid tradition of European commercial diplomacy*” ( Li, 2015) or praising the EU as an “ *independent power*” which constitutes an “ *important factor in multipolarisation.*” ( Wang, 2022b). In the economic realm, we also see a regular pattern of criticism, but this is mostly cautious and defensive, as for instance when Li Keqiang states that “ *putting the blame for excessive production capacity on China is neither objective nor fair, *” or when Foreign Minister Wang Yi picks up EU discourse on China as an economic competitor and then contradicts: “ *we are partners, not competitors*”. ( Li, 2017; Wang, 2019b).

In stark contrast to the fairly continuous pattern of discursive engagement that has emerged so far, there is a sudden jump in negative comments on the EU outside the economic sphere, which started in late 2020 so towards the end of the period covered here. All these comments relate to instances of perceived violations of Chinese sovereignty. For example, in 2020, President Xi Jinping reacted to EU criticisms on the human rights situation in Xinjiang and Hong Kong by stating that China “ *does not accept lecturing on human rights with a schoolmaster’s attitude*,” a phrase also picked up later by Wang Yi ( Anonymous, 2020; Wang, 2021b). The latter also accused “ *some people in Europe*” of “ *politicising economic issues*,” after the decision of the European Parliament to suspend deliberations on the CAI in reaction to Chinese countersanctions to the EU’s restrictive measures in relation to Xinjiang. ( Wang, 2021d).

This overview of Chinese leadership discourse across the first two periods in office of Xi Jinping suggests a few answers to the questions raised at the beginning of this section. Firstly, Chinese interest in the EU as an economic partner appears to remain unchanged, even in light of EU initiatives and debates that are seen as economically problematic by China. Critical representations do appear but are rare, and they are usually connected to specific developments that go against concrete Chinese interests. At the same time, European criticisms of China are countered by underlining the potential benefits of cooperation. This suggests a highly pragmatic discursive approach to economic engagement in the sense that questions of principle – as in the case of the provisions in the WTO accession agreement that relate to market economy status – are secondary as long as cooperation on the basis of negotiated compromise is possible.

A second conclusion, however, points to the limit of China’s pragmatism, namely any rhetoric or policy that is perceived as interfering with internal affairs, be it questions of domestic governance or territorial issues. This is of course nothing new and rhetorical tensions over human rights and Taiwan have been a regular feature of China-EU relations since the end of the Cold War. But that very continuity also means that change in Chinese attitudes on these matters is unlikely, which also entails that the recent momentum towards stronger cooperation with Taiwan will further complicate relations with Beijing. ( Gaenssmantel, 2025).

## Conclusions

Ultimately, we argue that the confluence of *Made in China 2025*, the *Dual Circulation Strategy* and the *BRI* has triggered a fundamental challenge to the EU’s commitment to a market-driven, liberal global economic system. China’s model is one of state capitalism where the innovation and growth of state-owned and major private enterprises are aligned with national strategic targets rather than purely commercial motives. In its external manifestations, as through strategic acquisitions, exports or investments that benefit from state subsidies and also non-reciprocal market access, this model challenges some core principles of European economic integration such as competition rules, transparency and reciprocity. The EU has considered this, at least partly, as a systemic challenge: it has moved beyond traditional trade defence mechanisms toward a comprehensive strategy of *de-risking.* As showcased above, this involves targeted new tools, e.g. the anti-subsidy regulation, but also a broader strategy of focusing heavily on building internal resilience, fostering domestic industrial champions and diversifying global supply chains in crucial areas such as critical raw materials and pharmaceutical inputs.

Therefore, it can be said that the EU’s future economic security and development will hinge on its ability to execute this pivot towards strategic autonomy while managing the complex interdependence of the world’s two largest trading blocs. This task becomes more complicated when we take into account that the interpretation and implementation level of de-risking widely varies between EU MSs ( Andersson & Lindbergh, 2024). A stronger European coordination on de-risking between EU MSs and EU institutions and an open discussion within the EU – and with the EU’s global partners – on the future priorities of de-risking would be beneficial to leverage the strategy at EU level. ( Casarini, 2024).

The momentum is right for such a leverage since China’s relations with the other key large developed industrial partners, notably the US but recently also Japan, have significantly deteriorated in the last years. China also needs the EU as a reliable economic partner – the economic, trade and supply chain dependencies work in both ways. This can explain the overall pragmatic tone of Chinese leadership that often goes hand-in-hand with underlining the potential benefits of EU-China economic cooperation (as evidenced by the analysis in the previous section). Bearing also in mind the limitations of China’s pragmatic approach when it comes to core political principles, the EU should take advantage of its current position and use its economic diplomacy toolset to re-negotiate key aspects of its economic links with China, such as moving more productive facilities of Chinese green technologies to Europe or enforcing a more open attitude from China towards European investments in certain economic sectors, ( Hemminga, 2025) possibly by way of a return to discussion on finalising and implementing CAI. In this way, the Chinese leadership need to show whether they truly intend to act upon their pragmatic discursive approach towards the EU with mutually beneficial trade deals in specific economic fields or they solely follow the declared goals of China’s national strategies of economic self-interest.

## Ethics and consent

Ethical approval and consent were not required for this research.

## Data Availability

The following datasets support the results and analysis of this article:
• Code summary • List of selected Chinese leadership speeches Both datasets are available at open repository Zenodo under the Creative Commons Attribution 4.0 International with DOI 10.5281/zenodo.18822892. Gaenssmantel, F. (2026). Reconnect China code summary and list of speeches (Version v01) [Data set]. Zenodo. https://doi.org/10.5281/zenodo.18822892

## Footnotes

^1^
Some scholars argue that China entered into its leadership phase at the global financial crisis in 2008, while others date this shift from the 18th CPC National Congress in 2012 when the new concepts for China’s economic diplomacy were put forth. See Li, X. (2022).

^2^

Even though the term was mentioned at least already in 2017 in context of China (see Chellaney, B. (2017, December 20). *China’s Creditor Imperialism.* Project Syndicate, https://www.project-syndicate.org/commentary/china-sri -lanka-hambantota-port-debt-by-brahma-chellaney-2017-12), there is an ongoing scholarly debate on the viability of the claim on a conscious Chinese debt strategy (see e.g. Jones, L., & Hameiri, S. (2020). *Debunking the myth of ‘debt-trap diplomacy’. How recipient countries shape China’s belt and road initiative.* Chatham House, or Brautigam, D. (2019). A critical look at Chinese ‘debt-trap diplomacy’: The rise of a meme. *Area Development and Policy, 5*(1), 1–14).

^3^
The project is led by Frank Gaenssmantel. Chen Fangjia, Li Haoqi, Yang Xiaotong and Hui Zhong provided valuable research assistance.
