# Supplementary material for: Policy and discursive shifts in China’s economic diplomacy amid rising geopolitical tensions
Source: Open Res Eur. 2026 Apr 13;6:109. [Version 1] doi: 10.12688/openreseurope.23140.1 (PMC13133616; doi:10.12688/openreseurope.23140.1)
Supplement: Peer review history

# Reviewer report: Reviewer response for version 1

**DOI:** 10.21956/openreseurope.25032.r72437

**Published:** 2026-04-30

**Authors:** Yanuar Rahmadan

**Affiliations:** 1 Universitas 17 Agustus 1945 Jakarta, Jakarta, Indonesia

Overall, the manuscript adheres to the current debate of the topics. Study design has appropriately used and fit to answer the research question. Although, there are a couple of things that may be included to give better pictures on the methods. For example, it may be helpful to specify the number of speeches and statements analysed in the study. The study uses official Chinese-language websites of the Chinese government bodies, but the sources could be specified more clearly. For instance, does the dataset include materials from the Ministry of Foreign Affairs website? Given the apparent reliance on MOFA sources in the references, it would be useful to provide a breakdown of the data: such as how many documents are drawn from the State Council website, the Ministry of Foreign Affairs, and other sources. This is crucial as I was unable to identify coding results derived from statements or speeches by senior officials from the relevant ministries. It may also be valuable to consider incorporating the role of the Chinese Communist Party into the analysis as Chinese' the evolving ideology of communist may also come to play (you may refer to article from Barsky, Vinogradov and Salitskii ( Dialectic Unity of the Domestic and Foreign Policy of ChinaOn the Centenary of the Communist Party of China - PMC ). Furthermore, it would be beneficial to include a summary of the coding results in the results section, enabling readers to gain an initial overview before engaging with the more detailed narrative discussion.

If applicable, is the statistical analysis and its interpretation appropriate?

Not applicable

Is the study design appropriate and is the work technically sound?

Yes

Is the work clearly and accurately presented and does it engage with the current literature?

Yes

Are the conclusions drawn adequately supported by the results?

Yes

Are sufficient details of methods and analysis provided to allow replication by others?

Yes

Are all the source data and materials underlying the results available?

Yes

Reviewer Expertise:

European Union external relations (trade); framing, discourse and content analysis.

I confirm that I have read this submission and believe that I have an appropriate level of expertise to confirm that it is of an acceptable scientific standard.

# Reviewer report: Reviewer response for version 1

**DOI:** 10.21956/openreseurope.25032.r72434

**Published:** 2026-04-24

**Authors:** Benjamin Daßler

**Affiliations:** 1 Ludwig-Maximilians-University Munich, Oettingenstr, Munich, Germany

Thank you for the opportunity to read this interesting article! This is a timely and well-structured paper that tackles an important question: has the EU's increasingly assertive stance toward China actually changed the way Chinese leaders talk about Europe? The answer that the authors arrive at, “essentially no”, is credible and (in the context of the author’s data analysis) well-grounded. I also found the contextual sections convincing. The five-stage periodisation of Chinese economic diplomacy and the treatment of Made in China 2025, the Dual Circulation Strategy, and the BRI as interlocking rather than separate instruments reflects command of the literature. The four-pillar de-risking framework is also explained clearly and accurately. The decision to work from official Chinese-language government sources rather than translated summaries is a justified methodological choice, and depositing the full dataset and coding framework on Zenodo is a model of transparency that I would encourage other authors to follow.

 That said, I have some framing, theory, and analytical suggestions which I hope to be helpful and constructive.

 The most important issue is the temporal gap. The contextual analysis draws on events through 2025, including Chinese export controls, countervailing duties on EVs, and the full de-risking legislative package. Yet the speech dataset ends in 2022, before de-risking was even formally announced. The paper's core claim is therefore tested against early signals of the EU's pivot, not its full enactment. I would encourage the authors to be much more explicit about this. The findings should be presented as a baseline for the pre-de-risking period, not as a definitive verdict on whether Chinese discourse has or has not shifted.

 Secondly, the paper documents that Chinese discourse did not change; it does not explain why. The structural argument that China needs the EU amid US-China rivalry is offered as a kind of background explanation, but it is never formally derived or tested. While I believe descriptive mapping of an under-studied phenomenon has value, the authors should be clear about what the paper is (a descriptive mapping of Chinese discourse over time) and is not doing in explanatory terms (theorizing changes/continuity) in this discourse.

 My third point is about how the results are presented. The Results section is informative and the illustrative quotations are well-chosen, but I cannot tell from reading it how many speeches contained negative versus positive representations of the EU, or how that balance changed year to year. A simple summary table or figure showing the distribution of key codes over time would make the finding of "discursive stability" more convincing. Right now it reads as a well-argued impression rather than a documented pattern.

 Fourth, I think the paper needs to grapple more seriously with what I would call the discourse-action gap. The authors rightly note that leadership speeches in China are carefully crafted consensus documents rather than genuine windows into policymakers' minds. But they do not fully confront the possibility that cooperative rhetoric toward the EU could coexist with or even serve to mask the systematic deepening of European dependencies through port acquisitions, the Digital Silk Road, and export controls on critical materials. I thought that the literature on strategic communication in authoritarian foreign policy would be a useful touchstone here.

 Finally, the speech selection criterion, which focuses on speeches where the EU is a substantive subject, likely introduces some upward bias toward cooperative language. The authors could acknowledge this explicitly.

 I have not independently verified the coding against the original Chinese-language speeches, nor have I accessed the Zenodo dataset directly to check its completeness. My assessment of the empirical findings relies on the authors' own characterisation and the quotations provided in the text.

If applicable, is the statistical analysis and its interpretation appropriate?

Not applicable

Is the study design appropriate and is the work technically sound?

Yes

Is the work clearly and accurately presented and does it engage with the current literature?

Yes

Are the conclusions drawn adequately supported by the results?

Yes

Are sufficient details of methods and analysis provided to allow replication by others?

Partly

Are all the source data and materials underlying the results available?

Yes

Reviewer Expertise:

International Relations; International Political Economy

I confirm that I have read this submission and believe that I have an appropriate level of expertise to confirm that it is of an acceptable scientific standard, however I have significant reservations, as outlined above.

# Reviewer report: Reviewer response for version 1

**DOI:** 10.21956/openreseurope.25032.r72430

**Published:** 2026-04-22

**Authors:** Mordechai Chaziza

**Affiliations:** 1 Ashkelon Academic College, Ashkelon, South District, Israel

This manuscript addresses an important and timely question: how China’s economic diplomacy, particularly toward the EU, has evolved discursively amid rising geopolitical tensions. The combination of discourse analysis with geoeconomic developments is promising, and the empirical base (coded leadership speeches, 2013–2022) is potentially valuable.

 However, in its current form, the manuscript does not yet meet the journal standards. The central limitation is the absence of a clearly articulated, theoretically grounded **“why”** argument. The paper remains largely descriptive and diagnostic, rather than explanatory. While the empirical material is solid, the analytical contribution is underdeveloped, and the causal mechanism linking geopolitical change to discursive continuity is insufficiently specified. With substantial revisions, particularly in theory-building, causal clarity, and analytical integration, the manuscript could become indexable in a high-ranking journal.

1. The manuscript asks whether discourse changed, but not why it did not change. The findings (discursive continuity despite structural change) are interesting but under-theorized.
2. The manuscript uses a broad definition of economic diplomacy but lacks engagement with International political economy theory, strategic narratives/discourse theory, and signaling and audience-cost frameworks.
3. The manuscript identifies patterns (pragmatism, continuity, limits at sovereignty issues) but does not sequence them into a causal mechanism.
4. The Introduction (and early sections) are overly long and policy-descriptive, especially on Made in China 2025, BRI, and EU de-risking instruments.
5. The coding strategy is described but lacks clarity about replicability, coding reliability, and justification of case selection.
6. The Results section reports patterns but often describes them rather than explains them.
7. The conclusion shifts into policy recommendations (an EU strategy), which dilute the theoretical contribution and feel disconnected from the discourse analysis.
8. Some sections are formulaic and repetitive; overuse of phrases like “this suggests that…”; occasional policy-report tone.
9. “Economic diplomacy,” “discursive representation,” and “strategic narratives” are used loosely.
10. Heavy reliance on policy and institutional sources. Limited engagement with top-tier academic literature.

Major Revise & Resubmit

If applicable, is the statistical analysis and its interpretation appropriate?

I cannot comment. A qualified statistician is required.

Is the study design appropriate and is the work technically sound?

Yes

Is the work clearly and accurately presented and does it engage with the current literature?

Yes

Are the conclusions drawn adequately supported by the results?

Yes

Are sufficient details of methods and analysis provided to allow replication by others?

Partly

Are all the source data and materials underlying the results available?

Yes

Reviewer Expertise:

China

I confirm that I have read this submission and believe that I have an appropriate level of expertise to confirm that it is of an acceptable scientific standard, however I have significant reservations, as outlined above.
